# Mathematical Modeling of the Influence of the Karman Vortex Street on Mass Transfer in Electromembrane Systems

**DOI:** 10.3390/membranes13040394

**Published:** 2023-03-30

**Authors:** Aminat Uzdenova, Anna Kovalenko, Evgeniy Prosviryakov, Makhamet Urtenov

**Affiliations:** 1 Department of Computer Science and Computational Mathematics, Umar Aliev Karachai-Cherkess State University, Karachaevsk 369202, Russia; 2 Department of Data Analysis and Artificial Intelligence, Kuban State University, Krasnodar 350040, Russia; 3 Department of Information Technologies and Control Systems, Ural Federal University the first President of Russia B. N. Yeltsin, 19 Mira St., Ekaterinburg 620049, Russia; 4 Department of Applied Mathematics, Kuban State University, Krasnodar 350040, Russia

**Keywords:** electromembrane system, mass transfer, spacers, Karman vortex street

## Abstract

In electromembrane systems, the transfer of ions near ion-exchange membranes causes concentration polarization, which significantly complicates mass transfer. Spacers are used to reduce the effect of concentration polarization and increase mass transfer. In this article, for the first time, a theoretical study is carried out, using a two-dimensional mathematical model, of the effect of spacers on the mass transfer process in the desalination channel formed by anion-exchange and cation-exchange membranes under conditions when they cause a developed Karman vortex street. The main idea is that, when the separation of vortices occurs on both sides in turn from the spacer located in the core of the flow where the concentration is maximum, the developed non-stationary Karman vortex street ensures the flow of the solution from the core of the flow alternately into the depleted diffusion layers near the ion-exchange membranes. This reduces the concentration polarization and, accordingly, increases the transport of salt ions. The mathematical model is a boundary value problem for the coupled system of Nernst–Planck–Poisson and Navier–Stokes equations for the potentiodynamic regime. The comparison of the current–voltage characteristics calculated for the desalination channel with and without a spacer showed a significant increase in the intensity of mass transfer due to the development of the Karman vortex street behind the spacer.

## 1. Introduction

Water is the most valuable resource for humanity and life in general. Today, however, half of the world’s river basins are under pressure beyond sustainable consumption [1]. The most precious water, clean drinking water, is virtually inaccessible to an estimated one billion people in developing countries.

In the future, the situation will worsen not only due to climate change [2,3], but also due to an increase in the global population to the expected 10 billion by 2050, as well as an increase in living standards with a change in consumption patterns [4,5,6].

In conditions where reliable drinking water supply is limited, the global water shortage for the world community becomes the number one problem [7]. The main solution to the problem of water shortage is desalination [7,8,9].

Economically and environmentally viable desalination methods are required to address water scarcity. Of these, electrodialysis is a globally recognized method for water purification, which demonstrates the potential to increase the overall efficiency of the process [10].

The efficiency of electrodialysis strongly depends on the hydrodynamics of the process, since the emergence of new high-performance membranes on the world market removes the kinetic restrictions associated with membranes and shifts the stage that determines the economic efficiency of desalination towards the liquid phase [11,12].

Recent studies show that there are two approaches based on solution flow control that can reduce the limitations of mass transfer from the electrolyte solution [12,13]. The first is the use of spacers, with which you can control the flow of the solution. We explored this approach in our study [14]. The second is the use of electroconvection under intense current regimes, the theory of which was developed in the fundamental works of I. Rubinshtein and B. Zaltzman [15,16,17]. However, in this case, such destructive processes arise and develop as the long-known reaction of the dissociation of water molecules, as well as the recently discovered breakdown of the space charge [18,19].

It is well known that in electromembrane systems (EMS), the transfer of ions near ion-exchange membranes causes concentration polarization, i.e., the formation of diffusion layers, which hinders mass transfer [20,21,22]. Spacers are used to mitigate the effect of concentration polarization and increase mass transfer [23,24,25,26,27,28,29,30].

From a hydrodynamic point of view, spacers reduce the thickness of the boundary layer by mixing the solution and creating a normal component of convective transport, so salt ions can reach the membranes faster and the current increases [30,31,32]. Concentration convection and thermal convection play an important role in wide desalination channels. The study of concentration convection and the mixing of complex liquids during thermal diffusion has recently become intensive [33,34]. To study various types of convection, new analytical and numerical methods are being developed [35,36]. In narrow channels, the main role in the overlimiting transfer of salt ions is electroconvection. Work [14,37,38,39,40,41,42,43,44,45] has been devoted to the mathematical modeling of the influence of spacers on mass transfer. These articles discuss the features of the influence of hydrodynamic flows due to the presence of spacers on the characteristics of mass transfer. Studies [46,47,48] found that electro-osmotic slip velocity or the applied electric field causes the shedding of vortices (or vortex street) in the channel containing spacers.

This article is devoted to the study of this problem and the possibility of the emergence of a developed unsteady Karman vortex street behind the spacer and its effect on the transfer of salt ions in the electrodialysis desalination channel. The characteristic forced flow velocity for the desalting channel of real electrodialysis desalting apparatuses, due to their considerable length, is of the order of 10 cm/s, and the channel width is 1–6 mm. If the transverse linear size of the spacer is one third of the channel width, then the Reynolds number for the spacer is of the order of 100. Thus, the Karman vortex street from the spacer in the desalination channel is formed and will be developed [49].

Due to its small size, the characteristic velocity for the experimental cell is much lower and is of the order of 0.5 cm/s. Therefore, other things being equal, the Reynolds number for the experimental cell is about 5, so the vortex street from the spacer does not appear in the experimental cell. Thus, a theoretical study of the effect of the Karman vortex street on the transport of salt ions is an urgent problem.

At low Reynolds numbers (below 100), the flow is stable. At Reynolds numbers of the order of 100, we have a developed unsteady Karman vortex street, when the separation of the vortices occurs on both sides in turn from the spacer, but the flow is still not turbulent, since the Reynolds number is much less than the critical value.

In this article, for the first time, a theoretical study of the effect of rectangular spacers on the process of mass transfer in the desalination chamber is carried out under conditions where they cause a developed unsteady Karman vortex street.

## 2. Materials and Methods

### 2.1. Mathematical Model

**Figure 1:**
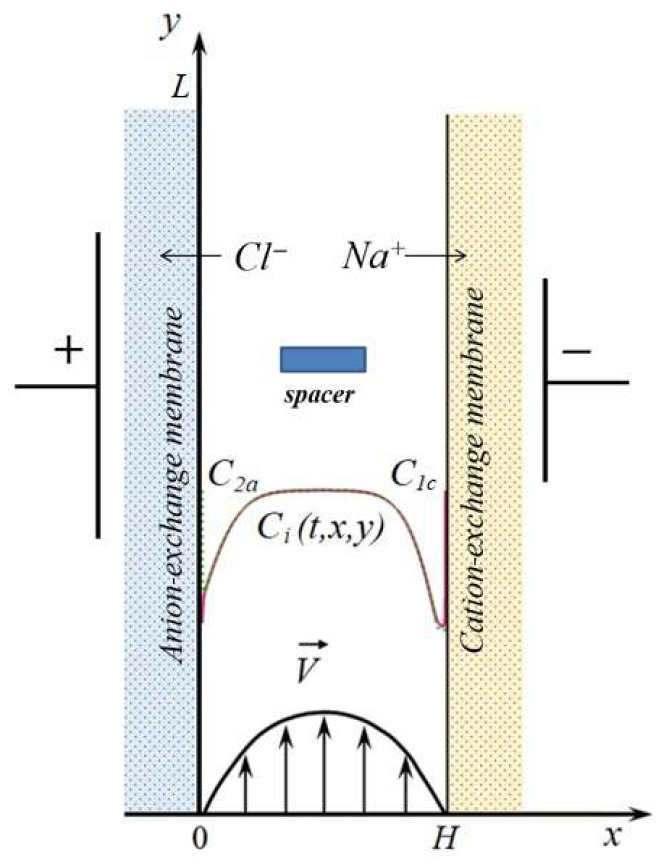
Scheme of a desalination channel formed between the anion- and cation-exchange membranes with a rectangular spacer. *H* and *L* are the width and length of the channel section. Schematically shown are the concentration profiles of cations (*C*_1_, solid line) and anions (*C*_2_, dotted line) in the desalination channel, the velocity of forced electrolyte flow at the inlet to the channel $\vec{V}$. $C_{2a}$ is the anion concentration at the solution/anion-exchange membrane interface; $C_{1c}$ is the cation concentration at the solution/cation-exchange membrane interface.

The scheme of the electrodialysis desalination channel with a spacer in the form of a rectangular bar placed at equal distances from the anion-exchange and cation-exchange membranes is shown in Figure 1. In this work, two-dimensional modeling is performed; i.e., the section of the desalination channel perpendicular to the surface of ion-exchange membranes is considered.

The unsteady transfer of binary electrolyte ions in the desalination channel under consideration, taking into account the forced flow and the development of electroconvection, is described by the following equations:

(1) $${\vec{j}}_{i}=\frac{F}{RT}z_{i}D_{i}C_{i}\vec{E}-D_{i}\nabla C_{i}+C_{i}\vec{V},\;i=1,2,$$

(2) $$\frac{\partial C_{i}}{\partial t}=-div\;{\vec{j}}_{i},\;i=1,2,$$

(3) $$\varepsilon_{r}\Delta \phi =-F(z_{1}C_{1}+z_{2}C_{2}),$$

(4) $$\vec{I}=F(z_{1}{\vec{j}}_{1}+z_{2}{\vec{j}}_{2}),$$

(5) $$\frac{\partial \vec{V}}{\partial t}+\left(\vec{V}\nabla \right)\vec{V}=-\frac{1}{\rho_{0}}\nabla P+v\Delta \vec{V}+\frac{1}{\rho_{0}}\rho \vec{E},$$

(6) $$div\;\vec{V}=0,$$

where the Nernst–Planck equations, shown in Equation (1), describe the flow of dissolved components (sodium $i=1↔Na^{+}$ and chlorine $i=2↔Cl^{-}$ ions, respectively, charge numbers of cations $z_{1}=1$, and anions $z_{2}=-1$), due to migration in an electric field, diffusion, and convection [50,51,52,53,54,55]; Equation (2) involves material balance equations; Equation (3) is the Poisson equation for the electric field potential, Equation (4) is the current flow equation, which means that the current flowing through the diffusion layer is determined by the flux of salt ions: $\varepsilon_{r}$ is the permittivity of the solution, F is the Faraday number, R is the universal gas constant, ϕ (V) is the potential, $C_{i}$ (mol/m^3^), ${\vec{j}}_{i}$ (mol/(m^2^s)), $D_{i}$ (m^2^/s), $\vec{I}$ (A/m^2^) is the concentration, flux, and diffusion coefficient of the *i*-th ion, the current density determined by the ion flux, and $\vec{V}$ (m/s) is the solution flow velocity; the Navier–Stokes equations shown in Equation (5) and the continuity equations for an incompressible fluid, Equation (6), describe the velocity field formed, among other things, under the action of a forced flow and a spatial electric force $\vec{f}=\rho \vec{E}\;$ (N/m^3^), where $\rho =F(z_{1}C_{1}+z_{2}C_{2})$(C/m^3^) is the space charge density, $\vec{E}=-\nabla \phi \;$ (V/m) is the electric field strength, $\rho_{0}$ (kg/m^3^) is the density of the solution, and v (m^2^/s) is the kinematic viscosity.

The boundary conditions of the model are formulated on the basis of the following assumptions: the surfaces of the ion-exchange membranes are assumed to be ideally selective and are impermeable to co-ions; the boundary concentration of counterions is determined by the exchange capacity of the membrane; the no-slip condition is specified for the velocity on the membrane surface; and the potentiodynamic mode of setting the electric field is considered when the potential jump increases linearly with time with a constant sweep rate. Thus, the boundary conditions have the form:

(a) At the electrolyte solution/anion-exchange membrane interface (x=0):

(7) $${\left.-\vec{n}\cdot \left(\frac{F}{RT}D_{1}C_{1}\nabla \phi -D_{1}\nabla C_{1}\right)\right|}_{x=0}=0,$$

(8) $$C_{2}\left(t,0,y\right)=C_{2a},$$

(9) $$-\vec{n}\cdot \vec{v}\left(t,0,y\right)=0,$$

(10) $$\phi \left(t,0,y\right)=d\cdot t,$$

where $\vec{n}$ is normal to the membrane surface, $C_{2a}$ is the anion concentration at the solution/anion-exchange membrane interface, and d (V/s) is the potential sweep rate.

(b) At the electrolyte solution/cation-exchange membrane interface (x=H):

(11) $${\left.-\vec{n}\cdot \left(\frac{F}{RT}D_{2}C_{2}\nabla \phi -D_{2}\nabla C_{2}\right)\right|}_{x=H}=0,$$

(12) $$C_{1}\left(t,H,y\right)=C_{1c},$$

(13) $$-\vec{n}\cdot \vec{v}\left(t,H,y\right)=0,$$

(14) $$\phi \left(t,H,y\right)=0,$$

(c) At the channel inlet (y=0):

(15) $$C_{i}\left(t,x,0\right)=C_{i0},i=1,2,\;\;\;\;\;\;\;\;\;\;C_{10}\left(t,x\right)-C_{20}\left(t,x\right)=0,$$

(16) $$\phi \left(t,x,0\right)=d\cdot t\cdot \frac{H-x}{H},$$

(17) $$V_{x}\left(t,x,0\right)=0,\;\;V_{y}\left(t,x,0\right)=6V_{0}\frac{x}{H}\left(1-\frac{x}{H}\right),$$

The potential distribution at the input was given by a linear function of time, as in Equation (16). With a constant distribution of concentrations at the inlet, Equations (15) and (16) follow from the fulfillment of Ohm’s law. The ion concentration and potential distribution are assumed to be given, so that the electrical neutrality condition is satisfied at the input, i.e.,$\;C_{10}\left(t,x\right)-C_{20}\left(t,x\right)=0$.

The flow rate of the solution $\vec{V}$ at the channel inlet will be considered distributed along the Poiseuille parabola, as in Equation (17), where $V_{0}$ is the average flow rate of the solution.

(d) At the channel outlet (y=L):

(18) $$-\vec{n}{\vec{j}}_{i}\left(t,x,L\right)=C_{i}\left(t,x,L\right)\vec{V}\left(t,x,L\right),-\vec{n}\nabla \phi \left(t,x,L\right)=0,-\vec{n}\vec{V}\left(t,x,L\right)=0.$$

The condition for the flow of ions (Equation (18)) assumes that salt ions are only carried out of the desalination channel due to the flow of the solution [56,57,58,59].

(e) At the boundaries of the spacer, the no-slip condition is applied, and the equality to zero of the ion fluxes normal to the boundaries and the components of the electric field strength. Thus, a non-conductive spacer is considered.
(f) The initial conditions are taken as consistent with the boundary conditions.

To assess the influence of the spacer and the Karman vortex street developing behind it on ion transport, let us compare the current–voltage characteristic (CVC) used for the desalination channel with and without a spacer, with the other parameters being the same. The current density is calculated by Equation (19) [57,58,59]:

(19) $$i_{av}\left(t\right)\approx \frac{1}{HL}\int_{0}^{H}\int_{0}^{L}I_{x}\left(t,s,y\right)dyds,$$

where $I_{x}\left(t,s,y\right)$ (A/m^2^) is calculated using a mathematical model according to Equation (4).

For the non-dimensionalization of the CVC, the limiting diffusion current is, according to Leveque Equation (20) [55]:

(20) $$i_{\lim}\left(t\right)\approx \frac{FDC_{0}}{H(T_{1}-t_{1})}\left[1.47{\left(\frac{H^{2}V_{0}}{LD}\right)}^{1/3}-0.2\right].$$

where $C_{0}$ is the initial concentration of the electrolyte solution, $D=D_{1}D_{2}\left(z_{1}-z_{2}\right)/\left(D_{1}z_{1}-D_{2}z_{2}\right)$(m^2^/s) is the diffusion coefficient of the electrolyte, and $T_{1}$ and $t_{1}$ are the transfer numbers of cation in the membrane and solution, respectively.

Thus, the mathematical model is a boundary value problem for the system of partial differential equations, Equations (1)–(18).

### 2.2. Numerical Implementation

Numerical solutions were found by the finite element method using Comsol Multiphysics^®^ 6.0 (www.comsol.com, COMSOL AB, Stockholm, Sweden) software package. The results presented below were obtained using a non-uniform unstructured triangular computational grid consisting of about 290,000 elements. The density of the mesh elements was increased near the solution/membrane boundaries: 2000 elements were set using the “Distribution” node. The mesh element density was increased near the solution/membrane boundaries with the Distribution node set to 2000 elements, as well as the Boundary Layers node. For the calculation with the spacer, the number of elements at its longitudinal (600 elements) and transverse (100 elements) boundaries was also increased.

For time-dependent calculations, a segregated node with implicit time-stepping method BDF (backward differentiation formulas) is used [60]. One segregated iteration consists of executing two segregated steps: in the first step, the concentration and potential are calculated; in the second step, the velocity and pressure are calculated. At each step, the multifrontal massively parallel sparse direct solver (MUMPS) method [60] is used.

The time step is automatically determined by the solver so that the requirement for the relative tolerance is met (its value was set equal to 10^−3^).

All calculations were carried out by using an Intel^®^ Core i7-4930K CPU (Intel de Costa Rica, La Ribera de Belen, Costa Rica).

### 2.3. Parameters Used in Computations

Below are the results for the following values of the system parameters: desalination channel width *H* = 6 mm, channel length *L* = 24 mm, average velocity of forced solution flow *V*_0_ = 4.8 cm/s, and initial solution concentration *C*_0_ = 0.01 mol/m^3^. It is assumed that the potential jump changes from 0 V to 1.7 V, with a sweep rate of *d* = 0.01 V/s, which ensures the quasi-stationarity of the CVC; that is, a further decrease in the sweep rate hardly changes the CVC at pre-limiting currents. The spacer dimensions are *h* = 0.6 mm and *l = 2* mm; it is placed at a distance of *7* mm from the entrance to the canal; *C*_2a_ = *C*_2c_ = *C*_0_. The transport numbers of cation in the cation-exchange membrane and in the solution are taken equal to $T_{1}=1$ and $t_{1}=0.395$, respectively. The Reynolds numbers of the channel and the spacer are equal to Re=V0H/ν≈323 and Res=V0h/ν≈108, respectively.

## 3. Results and Discussion

**Figure 2:**
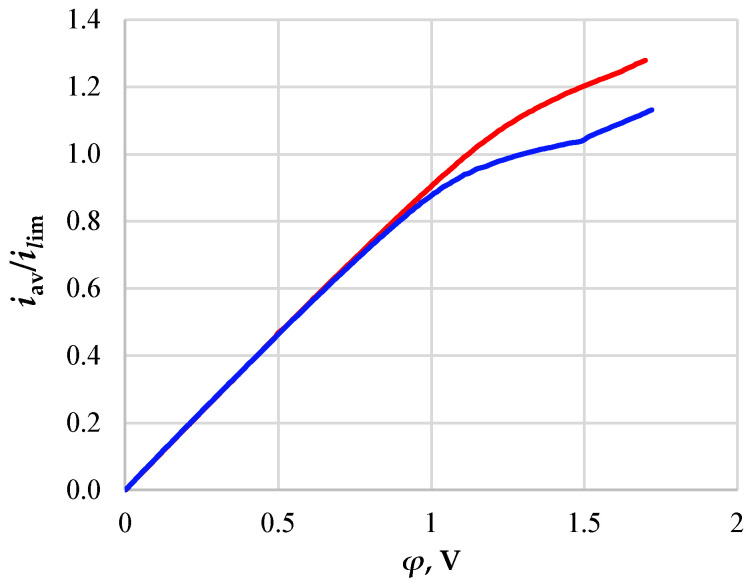
CVCs calculated for the desalination channels of an electrodialysis apparatus with NaCl solution without a spacer (blue line) and with a spacer insert in the center of the channel (red line).

Figure 2 shows the CVCs calculated for the desalination channel with (red line) and without (blue line) a spacer.

On the CVC for the desalination channel without a spacer, one can distinguish sections characteristic of the experimental curves, namely:

- The initial section of linear growth (at the potential jump up to 1 V), which is characterized by a rather high concentration of ions in the region near the membrane;
- The area of the “plateau” of the limiting current (between approximately 1 V and 1.5 V), which describes the saturation of the current corresponding to the almost complete depletion of ions at the membrane surface;
- The section of the overlimiting current due to the development of electroconvection (with the potential jump greater than 1.5 V).

**Figure 3:**
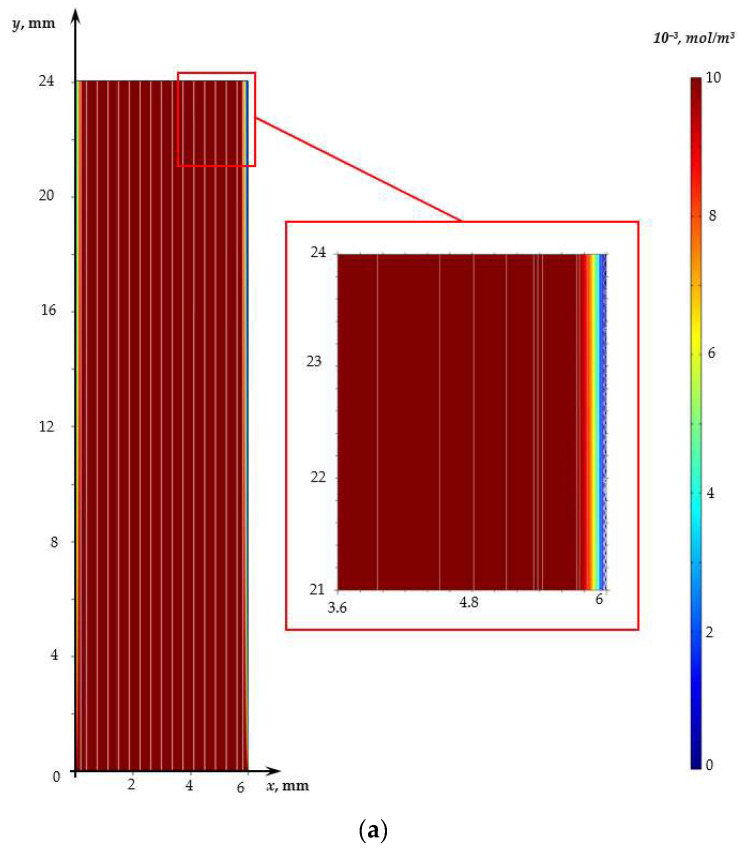

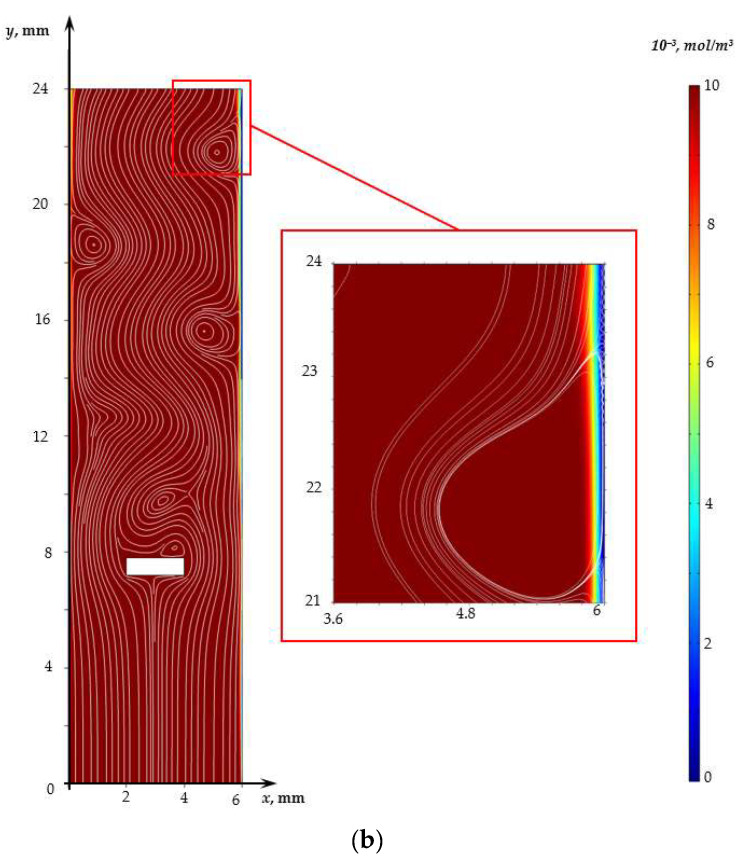
Electrolyte solution flow (white lines) and cation concentration distribution (shown in color) at time 170 s (φ = 1.7 V) for the channel without a spacer (**a**) and with the spacer (**b**) for NaCl solution.

In the considered range of potential jump values (from 0 to 1.7 V), the flow in the case without a spacer is irrotational in the entire desalination channel (Figure 3a), except for a narrow region (of the order of 20 μm) near the cation-exchange membrane in which electroconvective vortices are observed.

The CVC shape for a channel with a spacer differs from that described above for a channel without a spacer at the potential jump greater than 1V. Figure 2 shows the absence of an obvious current limit plateau for a channel with a spacer. This difference is fundamental and is associated with the non-stationary Karman vortex street developing behind the spacer (Figure 3b).

**Figure 4:**
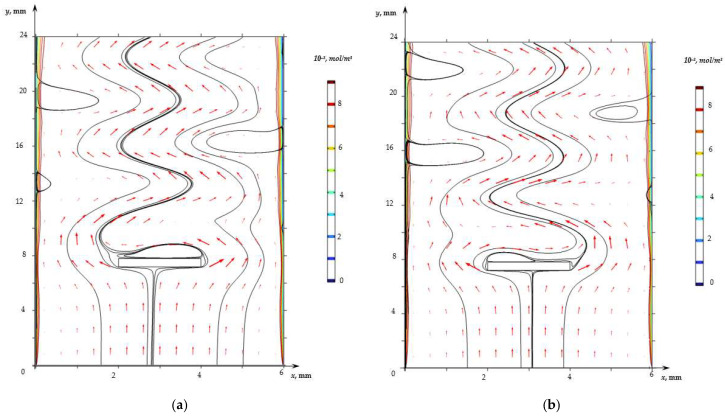
Electrolyte solution flow (black lines, arrows show the velocity field) and cation concentration distribution (colored level lines) for the desalination channel with spacer at time 170.65 s (**a**) and 170.70 s (**b**).

The visualization of the flow calculated on the basis of the model, Equations (1)–(18), for the indicated parameters showed the development of the unsteady Karman vortex street in the region behind the spacer (Figure 4, Video S1). Vortices are formed immediately by the spacer, the centers of which are alternately closer either to the left or to the right boundary of the spacer. Forced flow carries these vortices along the channel, causing a curvature of the flow lines of the solution; herewith, vortices are formed in the regions near the surfaces of the membranes, the dimensions of which reach 2 mm.

Calculations show that the vortex street in the case under consideration is formed in the first seconds and is preserved throughout the considered time interval. However, its effect on the mass transfer rate (the difference in CVCs) manifests itself at a potential jump greater than 1 V (Figure 2), when solution depletion regions form in the regions near the surface of both membranes and vortices supply the solution from the depth of the channel to these regions. It should be noted that in the case of calculations for the channel with the spacer, the electroconvective flow also develops at *φ* = 1.6 V. The thickness of the electroconvective vortex layer increases with the increasing potential jump (at *φ* = 1.7 V, it is about 10 µm; see Figure 3b).

**Figure 5:**
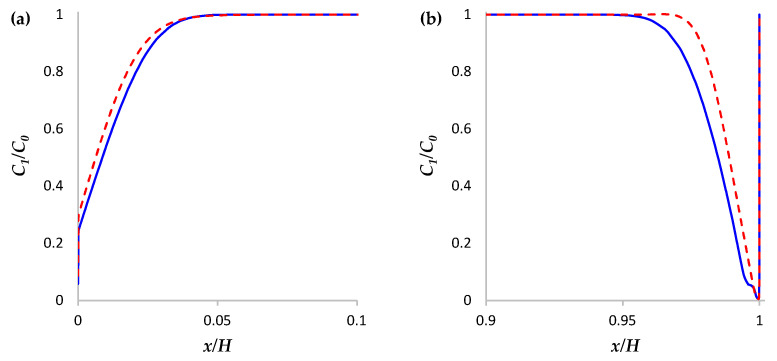
Concentration profiles of cations in the section *y* = 0.85 L calculated without a spacer (solid line) and with a spacer (dashed line) at the time point *t* = 170 s (**a**,**b**) regions near the surfaces of the anion- and cation-exchange membranes, respectively.

The vortices caused by the development of the Karman vortex street reach the surface of the membranes and affect the distribution of the ion concentration. Figure 4 shows that the thickness of the area of the desalinated solution at the locations of the vortices at a given moment (where the flow brings the “fresh” solution to the membrane surface) is less than in neighboring areas. Figure 5 shows the concentration profiles of cations in the cross section *y* = 0.85 L (regions near the surfaces of the anion- and cation-exchange membranes) calculated without a spacer (solid line) and with a spacer (dashed line) at time point *t* = 170 s. Figure 5 shows that the concentration of cations in the depleted diffusion layer near the surface of the anion- and cation-exchange membrane is higher in the case of the channel with a spacer due to the fact that the flow of the solution brings a more concentrated solution from the middle part of the channel to the diffusion layer near the ion-exchange membrane. At the same time, the periodicity of the change in the direction of the flow of the vortex street provides an increase in the electrolyte concentration near the surfaces of both membranes.

## 4. Conclusions

The main regularities of the effect of the Karman path formed behind a rectangular spacer on the transfer of salt ions in the desalination channel are determined taking into account electroconvection. It is shown that the developed unsteady Karman track formed from a spacer located in the core of the flow, where the concentration is at its maximum, ensures the flow of the solution from the core of the flow alternately into the depleted diffusion layers near the ion-exchange membranes, which, as a comparison of the CVC shows, leads to a significant increase in mass transfer.

This study is limited to calculations for one set of parameters specific to the electrodialysis desalination channel. In the future, extensive studies are planned to assess the influence of the Karman flow developing behind the spacer on the transfer of ions from the initial electrolyte concentration, the ratio of the spacer size to the channel width, and also to the linear velocity of the solution pumping.

## Data Availability

The data presented in this study are available on request from the corresponding author.

## Supplementary Materials

The following supporting information can be downloaded at: http://dx.doi.org/10.13140/RG.2.2.29590.22089, accessed on 1 March 2023, Video S1: KarmanVortexStreet.

## References

[1] Hoekstra A.Y., Wiedmann T.O. (2014). Humanity’s unsustainable environmental footprint. Science.

[2] Petersen L., Heynen M., Pellicciotti F. (2016). Freshwater Resources: Past, Present, Future. Int. Encycl. Geogr..

[3] Dinar A., Tieu A., Huynh H. (2019). Water scarcity impacts on global food production. Glob. Food Secur..

[4] Mekonnen M., Hoekstra A.Y. (2016). Four billion people facing severe water scarcity. Sci. Adv..

[5] Gude V.G. (2017). Desalination and water reuse to address global water scarcity. Rev. Environ. Sci. Bio/Technol..

[6] Djehdian L.A., Chini C.M., Marston L., Konar M., Stillwell A.S. (2019). Exposure of urban food-energy-water (FEW) systems to water scarcity. Sustain. Cities Soc..

[7] Jones E., Qadir M., van Vliet M.T., Smakhtin V., Kang S.M. (2019). The state of desalination and brine production: A global outlook. Sci. Total Environ..

[8] Ali A., Tufa R.A., Macedonio F., Curcio E., Drioli E. (2018). Membrane technology in renewable-energy-driven desalination. Renew. Sustain. Energy Rev..

[9] Tzanakakis V.A., Paranychianakis N.V., Angelakis A.N. (2020). Water Supply and Water Scarcity. Water.

[10] Doornbusch G., van der Wal M., Tedesco M., Post J., Nijmeijer K., Borneman Z. (2021). Multistage electrodialysis for desalination of natural seawater. Desalination.

[11] Golubenko D.V., Yaroslavtsev A.B. (2021). Effect of current density, concentration of ternary electrolyte and type of cations on the monovalent ion selectivity of surface-sulfonated graft anion-exchange membranes: Modelling and experiment. J. Membr. Sci..

[12] Kim B., Kwak R., Kwon H.J., Pham V.S., Kim M., Al-Anzi B., Lim G., Han J. (2016). Purification of High Salinity Brine by Multi-Stage Ion Concentration Polarization Desalination. Sci. Rep..

[13] Kim J., Kim S., Kwak R. (2021). Controlling ion transport with pattern structures on ion exchange membranes in electrodialysis. Desalination.

[14] Kovalenko A.V., Evdochenko E., Stockmeier F., Köller N., Uzdenova A., Urtenov M.A.K. (2021). Influence of spacers on mass transport in electromembrane desalination systems. J. Phys. Conf. Ser..

[15] Rubinstein I. (1991). Electroconvection at an electrically inhomogeneous permselective interface. Phys. Fluids A.

[16] Rubinstein I., Zaltzman B. (2000). Electro-osmotically induced convection at a permselective membrane. Phys. Rev. E.

[17] Zaltzman B., Rubinstein I. (2007). Electro-osmotic slip and electroconvective instability. J. Fluid Mech..

[18] Kovalenko A., Uzdenova A., Urtenov M. (2022). Theoretical Investigation of the Phenomenon of Space Charge Breakdown in Electromembrane Systems. Membranes.

[19] Kovalenko A.V., Wessling M., Nikonenko V.V., Mareev S.A., Moroz I.A., Evdochenko E., Urtenov M.K. (2021). Space-Charge breakdown phenomenon and spatio-temporal ion concentration and fluid flow patterns in over-limiting current electrodialysis. J. Membr. Sci..

[20] Uzdenova A., Kovalenko A., Urtenov M. (2022). Theoretical Analysis of Electroconvection in the Electrodialysis Desalination Channel under the Action of Direct Current. Membranes.

[21] Kovalenko A.V., Uzdenova A.M., Sukhinov A.I., Chubyr N.O., Urtenov M.K. Simulation of galvanic dynamic mode in membrane hydrocleaning systems taking into account space charge. Proceedings of the XV AIP International Scientific-Technical Conference “Dynamics of Technical Systems” (DTS-2019).

[22] Pismenskiy A., Urtenov M., Kovalenko A., Mareev S. (2015). Electrodialysis desalination process in conditions of mixed convection. Desalination Water Treat..

[23] Mareev S.A., Butylskii D.Y., Kovalenko A.V., Petukhova A.V., Pismenskaya N.D., Nikonenko V.V., Dammak L., Larchet C. (2016). Accounting for the concentration dependence of electrolyte diffusion coefficient in the Sand and the Peers equations. Electrochim. Acta.

[24] Długołȩcki P., Gambier A., Nijmeijer K., Wessling M. (2009). Practical potential of reverse electrodialysis as process for sustainable energy generation. Environ. Sci. Technol..

[25] Strathmann H. (2010). Electrodialysis, a mature technology with a multitude of new applications. Desalination.

[26] Sonin A.A., Isaacson M.S. (1974). Optimization of flow design in forced flow electrochemical systems, with special application to electrodialysis. Ind. Eng. Chem. Process Des. Dev..

[27] Balster J., Pünt I., Stamatialis D., Wessling M. (2006). Multi-layer spacer geometries with improved mass transport. J. Membr. Sci..

[28] Winograd Y., Solan A., Toren M. (1973). Mass transfer in narrow channels in the presence of turbulence promoters. Desalination.

[29] Kim Y., Walker W.S., Lawler D.F. (2011). Electrodialysis with spacers: Effects of variation and correlation of boundary layer thickness. Desalination.

[30] La Cerva M.L., Liberto M.D., Gurreri L., Tamburini A., Cipollina A., Micale G., Ciofalo M. (2017). Coupling CFD with a one-dimensional model to predict the performance of reverse electrodialysis stacks. J. Membr. Sci..

[31] Burmasheva N.V., Prosviryakov E.Y. (2020). Exact solution for stable convective concentration flows of a couette type. Comput. Contin. Mech..

[32] Burmasheva N.V., Prosviryakov E.Y. (2021). Exact Solution for Couette-Type Steady Convective Concentration Flows. J. Appl. Mech. Tech. Phys..

[33] Burmasheva N.V., Prosviryakov E.Y. (2020). On Marangoni shear convective flows of inhomogeneous viscous incompressible fluids in view of the Soret effect. J. King Saud Univ. –Sci..

[34] Burmasheva N.V., Privalova V.V., Prosviryakov E.Y. (2021). Layered Marangoni convection with the Navier slip condition. Sadhana-Acad. Proc. Eng. Sci..

[35] Ershkov S.V., Prosviryakov E.Y., Burmasheva N.V., Christianto V. (2021). Towards understanding the algorithms for solving the Navier–Stokes equations. Fluid Dyn. Res..

[36] Aristov S.N., Prosviryakov E.Y. (2016). A new class of exact solutions for three-dimensional thermal diffusion equations. Theor. Found. Chem. Eng..

[37] Gurreri L., Battaglia G., Tamburini A., Micale G., Ciofalo M. (2017). Multi-physical modelling of reverse electrodialysis. Desalination.

[38] Zhou C., Zhang H., Li Z., Wang W. (2016). Chemistry pumps: A review of chemically powered micropumps. Lab A Chip.

[39] Tadimeti J.G.D., Kurian V., Chandra A., Chattopadhyay S. (2016). Corrugated membrane surfaces for effective ion transport in electrodialysis. J. Membr. Sci..

[40] Vasil’eva V., Shaposhnik V., Grigorchuk O. (2001). Local mass transportduring electrodialysis with ion-exchange membranes and spacers. Russ. J. Electrochem..

[41] Kim I.H., Chang H.N. (1983). Experimental study of mass transportaround a turbulence promoter by the limiting current method. Int. J. Heat Mass Transf..

[42] Fischl D., Hanson K., Muller R., Tobias C. (1985). Mass transfer enhancement by small flow obstacles in electrochemical cells. Chem. Eng. Commun..

[43] Długołȩcki P., Dabrowska J., Nijmeijer K., Wessling M. (2010). Ion conductive spacers for increased power generation in reverse electrodialysis. J. Membr. Sci..

[44] Balster J., Stamatialis D., Wessling M. (2010). Membrane with integrated spacer. J. Membr. Sci..

[45] Kim B., Choi S., Pham V.S., Kwak R., Han J. (2017). Energy efficiency enhancement of electromembrane desalination systems by local flow redistribution optimized for the asymmetry of cation/anion diffusivity. J. Membr. Sci..

[46] Liang Y.Y., Fimbres Weihs G.A., Fletcher D.F. (2018). CFD study of the effect of unsteady slip velocity waveform on shear stress in membrane systems. Chem. Eng. Sci..

[47] Foo K., Liang Y.Y., Weihs G.A.F. (2019). CFD study of the effect of SWM feed spacer geometry on mass transfer enhancement driven by forced transient slip velocity. J. Membr. Sci..

[48] Foo K., Liang Y.Y., Tan C.K., Fimbres Weihs G.A. (2021). Coupled effects of circular and elliptical feed spacers under forced-slip on viscous dissipation and mass transfer enhancement based on CFD. J. Membr. Sci..

[49] Milton V.D. (1982). An Album of Fluid Motion.

[50] Urtenov M.K., Uzdenova A.M., Kovalenko A.V., Nikonenko V.V., Pismenskaya N.D., Vasil’eva V.I., Sistat P., Pourcelly G. (2013). Basic mathematical model of overlimiting transfer enhanced by electroconvection in flow-through electrodial-ysis membrane cells. J. Membr. Sci..

[51] Uzdenova A.M., Kovalenko A.V., Urtenov M.A.K. (2011). Mathematical Models of Electroconvection in Electromembrane Systems.

[52] Kwak R., Pham V.S., Lim K.M., Han J. (2013). Shear flow of an electrically charged fluid by ion concentration polarization: Scaling laws for electroconvective vortices. Phys. Rev. Lett..

[53] Nikonenko V.V., Mareev S.A., Pis’menskaya N.D., Uzdenova A.M., Kovalenko A.V., Urtenov M.K., Pourcelly G. (2017). Effect of electroconvection and its use in intensifying the mass transfer in electrodialysis (Review). Russ. J. Electrochem..

[54] Chubyr N.O., Kovalenko A.V., Urtenov M.A.K. (2012). Two-Dimensional Mathematical Models of Binary Electrolyte Transfer in Membrane Systems (Numerical and Asymptotic Analysis).

[55] Nikonenko V.V., Kovalenko A.V., Urtenov M.K., Pismenskaya N.D., Han J., Sistat P., Pourcelly G. (2014). Desalination at overlimiting currents: State-of-the-art and perspectives. Desalination.

[56] Urtenov M.K., Kovalenko A.V., Sukhinov A.I., Chubyr N.O., Gudza V.A. (2019). Model and numerical experiment for calculating the theoretical current-voltage characteristic in electro-membrane systems. IOP Conf. Ser. Mater. Sci. Eng..

[57] Kovalenko A.V., Urtenov M.K. (2020). Analysis of the theoretical CVC of electromembrane systems. E3S Web Conf..

[58] Gudza I.V., Urtenov M.K., Kovalenko A.V., Chubyr N.O. Analysis of the theoretical current-voltage characteristic in electromembrane systems. Proceedings of the Ion Transport in Organic and Inorganic Membranes.

[59] Kovalenko A.V. (2019). Mathematical Modeling of Transfer Processes in Electromembrane Systems. Ph.D. Thesis.

[60] Comsol Multiphysics Reference Manual. https://doc.comsol.com/6.0/doc/com.comsol.help.comsol/COMSOL_ProgrammingReferenceManual.pdf.

